# Comparison of the effects of drug-coated balloon and bare metal stent in the treatment of lower extremity arteriosclerosis obliterans

**DOI:** 10.12669/pjms.41.5.11520

**Published:** 2025-05

**Authors:** Yu Zhang, Kefan Wang, Lijing Zhou, Nan Guo, Qingyou Chen, Yuanyuan Zhai

**Affiliations:** 1 Yu Zhang, Department of Electrophysiology, Third Affiliated Hospital of Qiqihar Medical College, 27 Taishun Street, Qiqihar, Heilongjiang Province 161000, P.R. China; 2 Kefan Wang, Department of Electrophysiology, Third Affiliated Hospital of Qiqihar Medical College, 27 Taishun Street, Qiqihar, Heilongjiang Province 161000, P.R. China; 3 Lijing Zhou, Department of Second Resident Outpatient, General Hospital of Eastern Command, 305 Zhongshan East Road, Nanjing, Jiangsu Province 210000, P.R. China; 4 Nan Guo, Department of Electrophysiology, Third Affiliated Hospital of Qiqihar Medical College, 27 Taishun Street, Qiqihar, Heilongjiang Province 161000, P.R. China; 5 Qingyou Chen, Department of Electrophysiology, Third Affiliated Hospital of Qiqihar Medical College, 27 Taishun Street, Qiqihar, Heilongjiang Province 161000, P.R. China; 6 Yuanyuan Zhai, Department of Electrophysiology, Third Affiliated Hospital of Qiqihar Medical College, 27 Taishun Street, Qiqihar, Heilongjiang Province 161000, P.R. China

**Keywords:** Ankle-brachial index, Bare metal stent, Drug-coated balloons, Lower extremity arteriosclerosis obliterans, Pulse volume recording

## Abstract

**Objective:**

To compare the efficacy of drug-coated balloons (DCB) and bare metal stents (BMS) in treating lower extremity arteriosclerosis obliterans (LEASO).

**Methods:**

Clinical data of 83 patients who underwent LEASO angioplasty with DCB or BMS at the Third Affiliated Hospital of Qiqihar Medical College from July 2021 to January 2024 were retrospectively analyzed. Patients were divided into the DCB (n=43) and BMS (n=40) groups based on the treatment method. Changes in ankle-brachial index (ABI), pulse volume recording (PVR), vascular endothelial function indicators, incidence of complications, and patency rate were compared between the two groups.

**Results:**

Three months after the surgery, the ABI and nitric oxide (NO) levels in the DCB group were higher, while the upstroke time (UT), percentage of mean arterial pressure (%MAP), brachial-ankle pulse wave velocity (baPWV), and endothelin-1 (ET-1) levels were lower than those in the BMS group (P<0.05). There was no significant difference in the incidence of complications between the two groups (P>0.05). One year after the surgery, the patency rate of the DCB group was higher than that of the BMS group (P<0.05).

**Conclusions:**

DCB treatment for LEASO is superior to BMS in improving vascular function, protecting endothelial function, and achieving a one-year patency rate.

## INTRODUCTION

Lower extremity arteriosclerosis obliterans (LEASO) is a chronic progressive disease mainly caused by atherosclerosis of lower extremity arteries.^1^,^2^ LEASO patients often experience varying degrees of numbness, resting pain, and decreased skin temperature in the lower limbs. Skin ulcerations and necrosis may occur in more severe cases, often necessitating amputation.^1^–^3^ In recent years, with the continuous rise in the incidence of hyperlipidemia, diabetes, and hypertension, which are among the major risk factors of LEASO, safe and effective treatment of this disease has become a focus of substantial research.^4^,^5^

Currently, angioplasty using a bare metal stent (BMS) or drug-coated balloon (DCB) is the most commonly used procedure for treating LEASO.^6^,^7^ BMS acts as a scaffolding device that becomes epithelialized upon insertion and is integrated into the endothelium over time.^8^ DCB is a coronary device comprising a semi-compliant balloon catheter coated with antiproliferative agents that are delivered locally to the vessel wall during angioplasty.^9^ However, in the cases of challenging lesions and significant plaque burden, both methods are associated with a relatively high restenosis rate, which negatively impacts long-term prognosis.^10^–^14^ However, studies comparing the clinical efficacy of BMS and DCB in treating LEASO are still scarce.

Ankle-brachial index (ABI) and pulse volume map (PVR) are widely used as diagnostic and therapeutic tools for detecting and evaluating peripheral arterial disease.^15^,^16^ This study aimed to retrospectively analyze and compare the effectiveness of BMS and DCB in patients who underwent LEASO angioplasty by assessing ABI, PVR, and one-year postoperative patency rate.

## METHODS

This retrospective study included records of LEASO patients who underwent DCB or BMS at the Third Affiliated Hospital of Qiqihar Medical College from July 2021 to January 2024. Patients were divided into the DCB group and the BMS group according to different surgical methods.

### Ethical approval:

Our hospital’s ethics committee approved the study, No, Qi-2021-104, approval date, of May 20^th^, 2024.

### Inclusion criteria:

- Patients met the LEASO diagnostic criteria.^1^
- Rutherford Stages II-IV.
- The surgery was performed by the same surgeon team.
- Complete clinical data and follow-up for more than one year after surgery.

### Exclusion criteria:

- Patients with a history of previous surgical or endovascular procedures on the affected limb.
- Patients undergoing salvage stent implantation after DCB expansion.
- Patients with severe liver, liver, and kidney dysfunction.
- Patients with blood disorders (hemophilia a).
- Patients with combined lower limb necrosis and infection.

### Preoperative preparation:

The patient was given oral aspirin enteric coated tablets (100mg/day) (manufacturer: Bayer HealthCare Manufacturing S.r.l., Germany) for anticoagulation therapy. To improve microcirculation, patients received a 10μg/time/day injection of alprostadil (manufacturer: Bayer Schering Pharma AG; Germany). Patients with diabetes, hypertension, and hyperlipidemia were given symptomatic treatment aimed at lowering blood sugar, blood pressure, and lipid levels.

### Surgical procedure:

The patient was placed in a supine position, and routine disinfection and placement were performed. Lower limb local anesthesia was administered. The needle insertion point was located approximately 3cm above the inguinal ligament and extended to 4cm below the inguinal ligament. A single needle method was used to infiltrate and anesthetize the exposed area of the femoral artery surgery layer by layer in a diamond shape. The injection point was located at the medial epicondyle of the femur, and a diamond-shaped layer-by-layer infiltration anesthesia was applied upwards to the exposed area of the popliteal artery surgery on the knee joint (approximately 12 cm in length). After a successful puncture of the femoral/brachial artery using the Seldinger method, imaging of the affected limb was performed to clarify the condition.

Heparinization (heparine 0.5-0.6mg/kg body weight) of blood was performed. In cases of surgery time ≥ two hours, patients were supplemented with heparin at a dose of 50% of the initial dose. All patients first underwent regular balloon pre-dilation (maintained for 2-3 minutes). Patients in the DCB group underwent the insertion of DCB (manufacturer: Acotec; 3 mg/mm^2^ paclitaxel coating as an antiproliferative agent, carrier matrix: magnesium stearate; Beijing, China) for 3-5 minutes. Shallow femoral artery angiography was performed again to evaluate blood flow and to detect distal arterial embolism. The BMS group underwent stent placement for vascular recanalization, with the stent length exceeding 10mm in both the proximal and distal ends of the target lesion. Lesion angiography after stent implantation was performed to ensure an unobstructed lumen.

All patients received postoperative oral aspirin enteric coated tablets 100mg/d combined with clopidogrel (manufacturer: Sanofi; France) 75mg/d antiplatelet therapy for six months. After six months, patients transitioned to long-term oral aspirin enteric-coated tablets 100mg/d for antiplatelet therapy. Additionally, patients received long-term oral administration (10mg/d) of rosuvastatin calcium tablets (manufacturer: AstraZeneca; UK) after the surgery to stabilize the plaques.

### The following clinical characteristics of patients and relevant indicators before and three months after the surgery were collected:

- ABI and PVR were measured using the BP-203RPE III arterial sclerosis detector from Beijing Omron Medical Equipment Co., Ltd. Patients were instructed to lie flat. The phonocardiogram sensor, electrocardiogram clip, upper arm strap, and ankle strap were placed in their respective positions, and ABI and PVR parameters, such as brachial-ankle pulse wave velocity (baPWV), upstroke time (UT), percentage of mean arterial pressure (%MAP) were measured and recorded.
- Endothelial function: the levels of endothelin-1 (ET-1) and nitric oxide (NO) were measured in the serum of 4ml of venous blood by enzyme-linked immunosorbent assay.
- Complications, including infection, arterial embolism, and hematoma at the puncture site.
- Patients were followed for 12 months after the surgery and the patient’s patency was recorded.

### Statistical Analysis:

All data analysis was conducted using SPSS 25.0 software (IBM Corp, Armonk, NY, USA). The Shapiro-Wilk test was used to evaluate the normality of the evaluation data. Normal distribution data were represented by mean ± standard deviation, an independent sample t-test was used for inter-group comparison, and a paired t-test was used for intra-group comparison before and after the procedure. Non-normally distributed data were represented by a median and interquartile range, and the Whitney U test was used for inter-group comparison. The count data were represented by the number of cases, and compared using the Chi-square test. Kaplan Meier survival function analysis was used to plot the relationship between postoperative target lesion vascular patency rate and time for two groups of patients, and the Logrank test was used for comparative analysis. P<0.05 indicated a statistically significant difference.

## RESULTS

This study included a total of 83 LEASO patients, 48 males and 35 females. The age of the cohort ranged from 49 to 85 years, with an average of 64.9 ± 7.5 years. Patients were grouped based on the angioplasty method, with 43 cases in the DCB group and 40 in the BMS group. There was no significant difference in clinical characteristics between the two groups of patients (*P*>0.05) (Table-I).

**Table-I:** Comparison of clinical characteristics between two groups.

Characteristics	DCB group (n=43)	BMS group (n=40)	t/χ^2^/Z	P
Age (years), mean±SD	65.4±8.1	64.5±6.9	0.569	0.571
Male (yes), n (%)	23(53.5)	25(62.5)	0.690	0.406
BMI (kg/m^2^), mean±SD	23.7±2.9	23.1±2.7	1.094	0.277
*Rutherford classification, n (%)*				
I	7 (16.3)	5 (12.5)	2.518	0.284
II	23 (53.5)	28 (70.0)		
III	13 (30.2)	7 (17.5)		
Smoking (yes), n (%)	20(46.5)	23(53.5)	0.690	0.406
Diabetes (yes), n (%)	27 (62.8)	20 (50.0)	1.380	0.240
Hyperlipidemia (yes), n (%)	23 (53.5)	18 (45.0)	0.597	0.440
Hypertension (yes), n (%)	26 (60.5)	22 (55.0)	0.254	0.614
Stroke (yes), n (%)	11 (25.6)	8 (20.0)	0.366	0.545
Block length (cm), M(P25/P75)	8 (7-9)	8 (7.5-9.5)	-1.323	0.186

*Note:* DCB: drug-coated balloon; BMS: bare metal stent; SD: standard deviation; BMI: body mass index.

**Fig.1:**
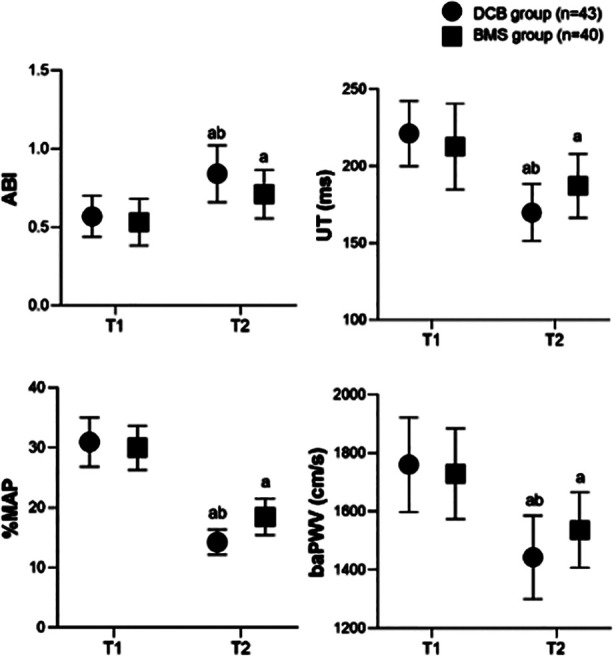
Comparison of ABI and PVR parameters between two groups; Compared to before surgery in this group, aP<0.05; Comparison with BMS group, bP<0.05; T1: preoperative; T2: three months after surgery; DCB: drug-coated balloon; BMS: bare metal stent; ABI: ankle-brachial index; PVR: pulse volume recording; UT: upstroke time; %MAP: percentage mean arterial pressure; baPWV: brachial-ankle pulse wave velocity.

Before the surgery, the two groups had no significant difference in the values of ABI, UT, % MAP, and baPWV (*P*>0.05). Three months after the surgery, the ABI of both groups increased, while UT, % MAP, and baPWV decreased compared to preoperative levels. At three months post-surgery, the ABI of the DCB group was significantly higher, while UT, % MAP, and baPWV were lower than those of the BMS group (*P*<0.05) (Fig.1).

**Fig.2:**
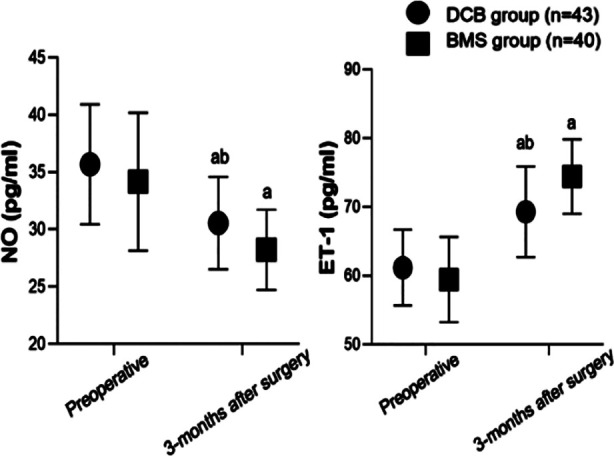
Comparison of endothelial function between two groups of blood vessels; Compared to before surgery in this group, aP<0.05; Comparison with BMS group, bP<0.05; DCB: drug-coated balloon; BMS: bare metal stent; NO: nitric oxide; ET-1: endothelin-1.

Before the surgery, serum NO and ET-1 levels were comparable between the two groups (*P*>0.05). Three months after the surgery, there was a significant decrease in NO and an increase in ET-1 in both groups compared to preoperative levels. The serum NO level in the DCB group was considerably higher than that in the BMS group, and the ET-1 level was significantly lower than that in the BMS group (P<0.05) three months post-surgery (Fig.2). There was no significant difference in the incidence of complications between the two groups (*P*>0.05) (Table-II).

**Table-II:** Comparison of incidence of complications between two groups.

Group	n	Infect	Arterial embolism	Hematoma at puncture site	Total incidence rate
DCB group	43	0 (0.0)	0 (0.0)	2 (4.7)	2 (4.7)
BMS group	40	2 (5.0)	1 (2.5)	2 (5.0)	5 (12.5)
*χ^2^*					0.793
*P*					0.373

**Fig.3:**
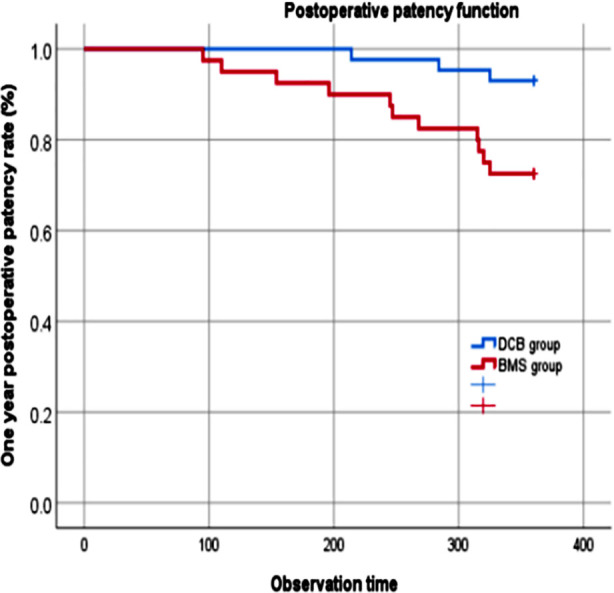
One-year postoperative patency of two groups; DCB: drug-coated balloon; BMS: bare metal stent.

After one year of postoperative follow-up, the incidence of restenosis was 7.0% (3/43) in the DCB group and 27.5% (11/40) in the BMS group, with a significant difference between the two groups (*χ^2^*=6.255; *P*=0.013). The Kaplan Meier survival function analysis was used to obtain the relationship between postoperative vascular patency rate and time in two groups of patients (Fig.3). The result of Logrank test showed a statistically significant difference in the postoperative vascular patency rate between the two groups of patients (*χ^2^*=6.372; *P*=0.012).

## DISCUSSION

This study compared the effects of DCB and BMS on ABI, PVR parameters, and endothelial function in patients who underwent LEASO angioplasty. Additionally, we compared the patency status of patients treated using DCB and BMS one year after the surgery. Our results indicated that using DCB for LEASO angioplasty was associated with significant advantages in treatment efficacy, improvement of vascular function, and patency rate. Hao Lisong et al.^17^ showed that compared with BMS, DCB has a better clinical effect in the treatment of lower limb atherosclerosis obliterans, and noted that the incidence of target lesion restenosis and malignant events was relatively low, which may be related to improved ABI and reduced expression of inflammatory factors.

A study by Zhang Kaixin et al.^18^ showed that using DCB was more efficient in treating lower extremity arterial occlusive disease (LEAOD) than BMS. It was associated with significantly improved arterial stenosis, increased ABI values, accelerated lower limb arterial blood flow recovery, and reduced rate of restenosis and malignant events. The results of this study are consistent with the above research, confirming the higher efficiency of DCB treatment for LEASO angioplasty. We believe it is possible that paclitaxel, which coats the balloon, is slowly released into the vascular wall and inhibits intimal hyperplasia, thereby improving late-stage patency.^12^–^15^,^17^,^18^ However, a study by Wang Peng et al.^19^ that compared the efficacy of DCB and BMS in treating LEASO detected no significant inter-group difference in the ABI values, as well as in the patency rate one year after the surgery, which contradicts our observations. This discrepancy in the results may be related to patient selection bias or different surgical teams having different surgical experiences.

ABI and PVR are important indicators for evaluating the degree of lower limb arterial occlusion.^15^ This study showed that the ABI and PVR levels in the DCB group three months after the surgery were higher compared to the BMS group. These results further indicate that DCB has a more significant effect on restoring lower limb arterial blood supply, which may be related to its ability to more effectively dilate narrowed blood vessels, restore normal vessel diameter, and improve hemodynamics.^20^,^21^ In addition, studies have shown that BMS implantation may cause changes in vascular elasticity and compliance due to the stimulation of the stent on the vascular wall. In contrast, DCB causes less damage to the vascular endothelium, reducing vascular wall fibrosis.^22^,^23^

This study also showed that at three months after surgery, the NO level in the DCB group was higher, and the ET-1 level was lower than that in the BMS group. NO has vasodilatory and platelet aggregation-inhibiting effects, while ET-1 has vasoconstrictive effects.^24^,^25^ The superior levels of NO and ET-1 in the DCB group indicate that DCB has a relatively small impact on endothelial function and significantly reduces postoperative vascular restenosis and thrombosis. We may speculate that this effect is mainly due to DCB causing less damage to the vascular endothelium. DSB, therefore, is beneficial for maintaining the normal function of vascular endothelial cells and is able to better regulate the balance of vascular contractile factors.^26^ In contrast, BMS may cause endothelial dysfunction due to foreign body stimulation, which may affect the secretion of NO and ET-1.^26^,^27^

The results of this study showed that the incidence of complications in the DCB group was only 4.7%, lower than that in the BMS group (12.5%). While the difference was not statistically significant, our results imply that using DCB for LEASO angioplasty is associated with a certain level of safety. Previous studies have also found that DCB treatment for LEASO was accompanied by relatively fewer complications, mainly because DCB causes less damage to the vascular wall, reducing the risk of inflammatory reactions and thrombosis.^23^,^28^

We show that after one year of postoperative follow-up, the incidence of restenosis in the DCB group (7.0%) was significantly lower than in the BMS group (27.5%), and there was a statistically significant difference in the postoperative vascular patency rate between the two groups of patients. Our results demonstrate that DCB has a greater advantage over BMS in terms of medium- and long-term patency. We assume that the uniform drug delivery on the surface of DCB, and the absence of polymers or residual metal foreign bodies considerably lower the inflammatory response of the vascular endothelium, thus decreasing the risk of thrombosis.^29^,^30^ Although the implantation of BMS can provide strong radial support for the continuity of the vascular wall, its presence as a permanent metal implant inevitably causes continuous mechanical stimulation to the blood vessel wall, leading to inflammatory response, excessive endothelial cell proliferation, platelet aggregation, total thrombus formation, and further stenosis.^29^–^31^

### Limitations:

Firstly, the study’s sample size was relatively small, which may have a certain impact on the generalizability of the results. Secondly, relatively short follow-up times did not allow us to assess long-term efficacy and potential late complications. Thirdly, excluding patients who underwent salvage stent implantation after DCB expansion resulted in some bias in the statistical results of complications. Finally, the impact of the two treatment methods on patient prognosis and quality of life remains to be verified. Future research can further expand the sample size and extend the follow-up time to more comprehensively evaluate the advantages and disadvantages of the two treatment methods.

## CONCLUSION

This study indicates that DCB for LEASO angioplasty is superior to BMS in improving vascular function, protecting endothelial function, and achieving a one-year patency rate. Further large-scale, multicenter, long-term follow-up studies are needed to further validate our conclusions and provide more reliable evidence for clinical treatment. Our findings may contribute to develop more personalized and optimized treatment plans for LEASO patients, improving their quality of life and prognosis.

### Author’s contributions:

**YZ:** Study design, literature search and manuscript writing. Revision,

**KW, LZ, NG, QC and YZ:** Were involved in data collection, data analysis, interpretation and critical review.

All authors have read and approved the final manuscript and are accountable for integrity of the study.
